# Elucidating the development of cooperative anode-biofilm-structures

**DOI:** 10.1016/j.bioflm.2024.100193

**Published:** 2024-03-25

**Authors:** Edina Klein, René Wurst, David Rehnlund, Johannes Gescher

**Affiliations:** a Institute of Technical Microbiology, University of Technology Hamburg, Hamburg, Germany; b Department of Chemistry – Ångström Laboratory, Uppsala University, Box 538, SE-751 21, Uppsala, Sweden

## Abstract

Microbial electrochemical systems are a highly versatile platform technology with a particular focus on the interplay of chemical and electrical energy conversion and offer immense potential for a sustainable bioeconomy. The industrial realization of this potential requires a critical focus on biofilm optimization if performance is to be controlled over a long period of time. Moreover, the aspect and influence of cooperativity has to be addressed as many applied anodic bioelectrochemical systems will most likely be operated with a diversity of interacting microbial species. Hence, the aim of this study was to analyze how interspecies dependence and cooperativity of a model community influence the development of anodic biofilms. To investigate biofilm activity in a spatially resolved manner, a microfluidic bioelectrochemical flow cell was developed that can be equipped with user-defined electrode materials and operates under laminar flow conditions. With this infrastructure, the development of single and co-culture biofilms of the two model organisms *Shewanella oneidensis* and *Geobacter sulfurreducens* on graphite electrodes was monitored by optical coherence tomography analysis. The interdependence in the co-culture biofilm was achieved by feeding the community with lactate, which is converted by *S. oneidensis* into acetate, which in turn serves as substrate for *G. sulfurreducens*. The results show that co-cultivation resulted in the formation of denser biofilms than in single culture. Moreover, we hypothesize that *S. oneidensis* in return utilizes the conductive biofilm matrix build by *G. sulfurreducens* for direct interspecies electron transfer (DIET) to the anode. FISH analysis revealed that the biofilms consisted of approximately two-thirds *G. sulfurreducens* cells, which most likely formed a conductive 3D network throughout the biofilm matrix, in which evenly distributed tubular *S. oneidensis* colonies were embedded without direct contact to the anode surface. Live/dead staining shows that the outermost biofilm contained almost exclusively dead cells (98 %), layers near the anode contained 45–56 % and the entire biofilm contained 82 % live cells. Our results exemplify how the architecture of the exoelectrogenic biofilm dynamically adapts to the respective process conditions.

## 1 Introduction

Most known microorganisms rely on soluble, intracellularly available electron acceptors, such as oxygen, to enable their metabolism. Exoelectrogenic bacteria, however, have the unique ability to transfer respiratory electrons to insoluble extracellular electron acceptors, such as iron oxides, which are present in their natural habitat. In a synthetically created environment, such as a bioelectrochemical system (BES), this ability can be harnessed by using a solid-state anode as an inexhaustible extracellular terminal electron acceptor, thereby utilizing the microorganisms as biocatalysts for the conversion of chemical to electrical energy. The most efficient way of biocatalysis in these bioelectrochemical systems was revealed to be a biofilm of exoelectrogenic organisms that is directly formed on the electrodes [1].

Anode biofilms do not necessarily have to consist of a single cell layer that is in direct contact with the electrode surface. Some anode-respiring organisms like *Geobacter* species have developed extracellular conductive structures that enable the transfer of electrons over a distance of several micrometers. These structures can consist of outer membrane vesicles [2,3] or so-called nanowires which are composed of conductive monomers which form into 10–20 μm long structures [4,5]. The conductivity of these elements is based on *c*-type cytochromes and/or other proteins engaging aromatic amino acids for subunit to subunit bridging electron transfer [[6], [7], [8], [9], [10]]. In most model organisms for extracellular electron transfer, a network of *c*-type cytochrome proteins is used to transfer respiratory electrons from the cytoplasmic membrane to the cell surface [[11], [12], [13], [14], [15]]. Since the position of cytochromes in the outer membrane is dynamic and most cytochrome proteins of the model organisms studied so far operate in overlapping redox potential windows, electrons can be transferred not only from one cell to an electron acceptor, but also from one cell to a neighboring cell [4,16,17]. The direction of electron transfer is based on the position of the terminal electron sink which is the anode. Anode biofilms thus form a dynamic electron transfer network in which the transfer is based on a direct cell-electrode interaction for the cell layer that is in direct contact with the electrode, as well as on a cell-nanowire/vesicle-electrode interaction and a multicell interaction [4,6,16,17].

Usually, the electron donor of the cells diffuses into the biofilm from the bulk phase [18]. On the other hand, kinetics of electron transfer to the anode will decrease with increasing distance to the electrode [19]. Hence, electron transfer kinetics and electron donor availability have opposite vectoral directions. The diffusion of electron donor into the biofilm will mostly depend on its concentration, the diffusion coefficient of the biofilm and the flow velocity over the biofilm surface. The electron transfer will depend mostly on the conductivity of the EPS matrix as well as the metabolic state of the cells. Several studies aimed at elucidating factors limiting activity of anode biofilms [18,[20], [21], [22], [23], [24], [25]]. Notably, imaging of biofilm dimensions and correlating this with measured current densities can give valuable information. Hence, fluorescence microscopy as well as optical coherence tomography (OCT) studies were conducted with mixed environmental inocula and in bioelectrochemical flow cell systems of varying formats [[25], [26], [27], [28], [29], [30], [31], [32], [33], [34], [35], [36]]. In several of these studies, model presumptions were corroborated suggesting that the formation of proton gradients from anode to bulk phase will limit biofilm activity. Also, it was highlighted that electron donor availability can have a tremendous impact as well. With regards to biological activity in different biofilm depths, there is so far no clear result within past research. While some studies revealed a live outer layer with a dead zone close to the anode [28,34,35] others reported the opposite [26,29,33,37]. Another recent analysis using a flow cell and an environmental inoculum suggested that most metabolic activity would be in the center of 42.5–60 μm thick anode biofilms [36]. These varying observations might be a result of different conditions used in the studies. In particular, the use of flow-through reactors in comparison to batch systems is likely to have had an influence. This presumably also applies to the structure of the biofilm, which will also change according to flow conditions. In general, it seems reasonable to conduct experiments in flow-through systems, since the majority of potential industrial applications of BES, such as wastewater treatment or anode assisted fermentation, will take place as continuous processes under flow-through conditions. Additionally, electrode materials should be chosen close to biotechnological applications, if optimal biofilm properties for bioelectrochemical processes are being evaluated, since the electrode surface will affect biofilm formation and structure as well.

This study reports on the development of a microfluidic bioelectrochemical flow cell which can be equipped with user-defined electrode materials and is characterized by laminar flow conditions. The system was constructed for use with automated robotic OCT-analysis. Using this infrastructure, we show the development of single and co-culture biofilms of the two model organisms *Shewanella oneidensis* and *Geobacter sulfurreducens* on graphite electrodes. *S. oneidensis* is the best understood model organism regarding extracellular electron transfer and the ability to thrive with insoluble electron acceptors. Still, the achievable electron transfer rates with these electron acceptors are several folds lower compared to the other prominent model organism for extracellular electron transfer – *G. sulfurreducens*. Nevertheless, the aerotolerant to obligate anaerobic metabolism of *G. sulfurreducens* and its poor genetic accessibility limit its application to some extent. *S. oneidensis*, on the other hand, has a high genetic accessibility and a facultative anaerobic metabolism, which qualifies it as an appropriate candidate for biotechnological applications for instance in anode-assisted fermentations [38,39].

Using in-depth OCT-analysis together with chronoamperometric control allowed to draw clear conclusions regarding biofilm activity development for these two model organisms and the correlation between current density and biofilm structure. The here developed system was used also to analyze how the structure and functionality of a co-culture biofilm of the two organisms would differ from single species biofilms. Surprisingly, the analyses suggest not only structural changes of the biofilms as a result of co-culturing but also direct electron transfer between *S. oneidensis* and *G. sulfurreducens*. Due to these results, we hypothesize that the conductive biofilm matrix developed by *G. sulfurreducens* cells form a conductive network connected by nanowires and *S. oneidensis* in return most likely utilizes this conductive biofilm matrix for direct interspecies electron transfer (DIET) to the anode.

## 2 Materials and methods

### 2.1 Flow cell system

The polydimethylsiloxane (PDMS) microfluidic reactors with a straight channel design were manufactured as recently described [40]. Electrode material was a composite of graphite and polypropylene (PPG 86) with an electrical conductivity of 60.15 S cm^−2^ (Eisenhuth GmbH & Co. KG, Osterode am Harz, Germany). The material was corundum blasted to generate a rougher surface (Ra = 1.43 μm; Rz = 12.1 μm). Standard cannulas (B. Braun, Melsungen, Germany), BD Connecta™ micro-valves (Becton Dickinson, Franklin Lakes, New Jersey, USA) and luer lock fittings were used for connection to the medium supply. Media were supplied to the reactors via silicone tubings (Carl Roth, Karlsruhe, Germany) and for inoculation fluororubber tubings (Cole‐Parmer, Vernon Hills, IL, US) were used. The tubes had an inner diameter of 1.5 mm. Constant medium supply with a flow rate of 4 mL h^−1^ was assured using Reglo ICC peristaltic pumps (ISMATEC Industry Solutions GmbH, Grevenbroich, Germany). Prior to inoculation, the entire system was flushed with 70% ethanol and afterwards equilibrated with medium for at least 12 h.

### 2.2 Preparation of the tailor-made Ag/AgCl reference electrodes

The reference electrodes were prepared electrochemically at room temperature. For this purpose, a 3 cm long piece of silver wire (diameter: 0.8 mm; ChemPur, Karlsruhe, Germany) was cleaned by rinsing it with absolute ethanol. The dry silver wire, surrounded by a fine platinum mesh, was immersed in a beaker containing 0.1 M degassed hydrochloric acid. The surface of the beaker was flushed with nitrogen. Subsequently, the silver wire served as working electrode and the platinum mesh as counter electrode. A commercially available Ag/AgCl electrode was used as a reference. To equilibrate the electrochemical system, open circuit voltage (OCV) was first applied for 5 min and then an overpotential of +50 mV (vs. OCV) was applied for 50 min. The Ag/AgCl electrode thus prepared was dried after preparation. A freshly prepared reference electrode was utilized for each anode reactor.

### 2.3 Strains and growth conditions

*Shewanella oneidensis* MR-1 wild type strain [41] was pre-grown in lysogeny broth (LB) medium [42] overnight at 30 °C and 160 rpm. All bioelectrochemical experiments were conducted in minimal medium according to Dolch et al. [43] and the overnight precultures for the bioelectrochemical experiments were grown in this medium as well. The medium contained 0.42 g l^−1^ KH_2_PO_4_, 0.22 g l^−1^ K_2_HPO_4_, 0.2 g l^−1^ NH_4_Cl, 0.38 g l^−1^ KCl, 0.36 g l^−1^ NaCl, 1.8 g l^−1^ NaHCO_3_, 0.5 g l^−1^ Na_2_CO_3_, 0.213 g l^−1^ MgCl_2_ * 6H_2_O, 0.1 g l^−1^ casamino acids, 1 mL selenite tungstate solution (0.5 g l^−1^ NaOH, 3 mg l^−1^ Na_2_SeO_3_, 4 mg l^−1^ Na_2_WO_4_ * 2H_2_O), and 10 mL l^−1^ NB trace mineral solution [44,45]. The pH was adjusted to 7. The media were autoclaved, purged with 80% N_2_/20% CO_2_ gas and complemented with 10 mL L^−1^ vitamin solution (medium 141; German Type Culture Collection, DSMZ), 0.2 mM sodium ascorbate and 0.4 mM CaCl_2_ * 2H_2_O. *S. oneidensis* cells were transferred into this minimal medium containing 40 mM fumarate (electron acceptor) and 70 mM Na-D,l-lactate (electron donor) and grown overnight at 30 °C. *G. sulfurreducens* PCA wild type strain [46] was pre-grown at 30 °C for around 48 h in medium containing 15 mM sodium acetate, 40 mM fumarate and 0.1 % yeast extract (w/v). Cells were washed with washing buffer (0.42 g l^−1^ KH_2_PO_4_, 0.22 g l^−1^ K_2_HPO_4_, 0.2 g l^−1^ NH_4_Cl, 0.38 g l^−1^ KCl, 0.36 g l^−1^ NaCl) purged with 80% N_2_/20% CO_2_ gas. For co-culture experiments a suspension of *S. oneidensis*/*G. sulfurreducens* (10:1) was prepared from the overnight cultures with an optical density of 2.0 at 600 nm. An excess of *S. oneidensis* cells was inoculated, as *G. sulfurreducens* is dependent on the acetate supply from *S. oneidensis*. Cell suspension for single culture experiments were conducted similarly and the optical densities of these inocula were 2.0 at 600 nm. Inoculation was carried out with a L160 syringe pump (Landgraf Laborsysteme HLL GmbH, Langenhagen, Germany) for 2 h at a flow rate of 2 mL h^−1^ via a separate side port of the microfluidic reactor (Fig. S1). During inoculation, medium was supplied via the front port with a flow rate of 2 mL h^−1^ and after inoculation the flow rate of the medium was restored to 4 mL h^−1^.

### 2.4 Optical coherence tomography (OCT)

Mesoscopic structures of biofilms were monitored using optical coherence tomography (OCT). OCT images were captured using a Ganymede™ spectral domain system (GAN611C1-SP1, Thorlabs GmbH, Dachau, Germany). Parameters were chosen as described [47] and datasets were processed with (Fiji Is Just) ImageJ version 2.1.0/1.53 [48]. To describe and quantify the biofilms, the acquired data sets were processed in several sequential steps. Biofilm volume was calculated by correlating the number of pixel signals with the voxel size of the images. Height maps of the anodes were generated based on the process routine developed by Wagner and Horn [49] and coverage was determined based on these height maps. The porosity of the biofilms was determined by correlating the actual height h_real_ with a theoretically calculated height h_theoretical_ from the biovolume (100% density) using the following formula:

Formula 1 $$\frac{h_{real}-h_{theoretical}}{h_{real}}\times 100$$

### 2.5 Live/dead cell imaging

For live/dead staining of biofilms cultured in microfluidic flow reactors, the reactors were washed with phosphate buffered saline (PBS; 136.9 mM NaCl, 2.7 mM KCl, 10.1 mM Na_2_HPO_4_, 1.8 mM KH_2_PO_4_). This solution and all subsequent solutions were pumped through the reactors for 20 min in the dark at a flow rate of 4 mL h^−1^ using a syringe pump (LA160, Landgraf Laborsysteme HLL GmbH, Langenhagen, Germany). For the staining solution, 4.5 μL of propidium iodide solution (20 mM in dimethyl sulfoxide) and 3 μL of Syto 9 (5 mM in ddH_2_O; both from Invitrogen AG, Carlsbad, CA, US) were added to 3 mL of PBS. Finally, the reactors were washed again with PBS. Microscopic imaging was performed using a Leica DM5500B microscope, a Leica K5-14401820 camera, and Leica Application Suite X software version 3.8.0.26413 (Leica Microsystems, Wetzlar, Germany). First, images of the biofilms were taken with a dip-in objective (HC APO L U–V–I 63x/0.90 WATER UV, Leica Microsystems, Wetzlar, Germany) by carefully removing the electrode from the cultivation channel and placing it in a Petri dish containing PBS. To determine the amount of metabolically active cells compared to the cells with damaged membrane (dead cells) within the biofilm matrix, the biofilm was peeled off the electrode using PBS and a pipette. The biofilm was resuspended by pipetting up and down, transferred to a microscope slide and photographed using the N PLAN 100x/1.25 OIL objective (Leica Microsystems, Wetzlar, Germany). The electrode was placed in a Petri dish containing PBS and the remaining biofilm was visualized with the dip-in objective. To assess the viability of layers of biofilm closer to the anode, the biofilm was removed from the electrode with a scalpel. The biofilm was placed upside down on a microscope slide and a coverslip was placed on the former lower part of the biofilm. Images were taken with the N PLAN 100x/1.25 OIL objective (Leica Microsystems, Wetzlar, Germany) using the L5 ET (k) and Y3 ET (k) filter cubes from Chroma Technology Corporation (Bellows Falls, VT, USA). The remaining biofilm on the anode was visualized in the same way as for the other electrode. The percentage of dead and live cells was determined using (Fiji Is Just) ImageJ version 2.1.0/1.53 [48]. For this purpose, the images were converted to 8 bits, binarized with an appropriate threshold (0–255) and the percentages of live and dead cells were determined using Formula 2, Formula 3, respectively.

Formula 2 $$\frac{{area}_{live}}{{area}_{live}+{area}_{dead}}\times 100$$

Formula 3 $$\frac{{area}_{dead}}{{area}_{live}+{area}_{dead}}\times 100$$

### 2.6 Fluorescence *in situ* hybridization (FISH)

Since FISH protocols are based on the fixation of cultured cells, this type of experiment was performed as an endpoint analysis after 10 days of cultivation. An automated FISH procedure was performed using the probes SHEW227 (5'- [6-FAM]-AGC TAA TCC CAC CTA GGT WCA TC -3′) [50] and GEO2 (5' [Cyanine 3]-GAA GAC AGG AGG CCC GAA A -3′), The latter additionally requires two auxiliary oligonucleotides, HGEO2-1 (5′- GTC CCC CCC TTT TCC CGC AAG A -3′) and HGEO2-2 (5′- CTA ATG GTA CGC GGA CTC ATC C -3′) [51]. Cells were counterstained with 4′, 6-diamidino-2-phenylindole (DAPI; Merck KGaA, Darmstadt, Germany). The entire FISH procedure was performed within the microfluidic reactor by pumping the individual solutions through the biofilm directly after the end of the experiment. For this purpose, all solutions were connected to active 2/2 normally closed valves (SMV-2R–BN1F; Takasago Kōryō Kōgyō, Tokyo, Japan). The valves were controlled via a custom-built control unit. Via Y-branches, all solutions were brought together in a single outlet tube, which was connected to a REGLO ICC peristaltic pump (ISMATEC Industry Solutions GmbH, Grevenbroich, Germany). The solutions were delivered to the microfluidic reactor at 4 mL h^−1^. A more detailed description of the FISH protocol can be found in S1.

Images were acquired using the LSM 800 confocal laser scanning microscope (CLSM) with a Plan-Apochromat 63*x*/1.40 Oil DIC M27 objective (Carl Zeiss, Oberkochen, Germany). The ratios of *S. oneidensis* and *G. sulfurreducens* cells were determined using (Fiji Is Just) ImageJ version 2.1.0/1.53 [48]. For this purpose, the images were converted to 8 bits, binarized with an appropriate threshold (0–255) and the percentages of the two species were determined using Formula 2, Formula 3, respectively.

**2.7 Development of a multiparallel platform for automated analysis of electrode biofilms under laminar flow conditions.**

**Fig. 1:**
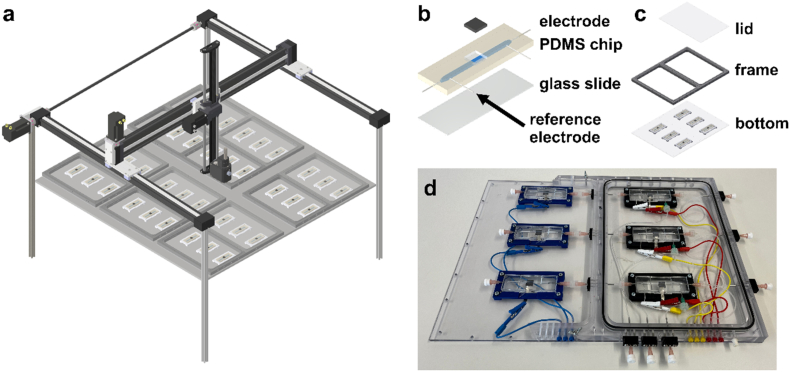
**An automated platform for electroactive biofilm cultivation and analysis.** The microfluidic cultivation platform allows up to six triplicates to be operated simultaneously for continuous cultivation of biofilms (**a**). By integrating an optical coherence tomograph into a gantry robot, biofilm growth can be studied *in situ*. The microfluidic cultivation chips (**b**) are made of polydimethylsiloxane (PDMS) and the channel is sealed by plasma bonding to a glass slide. A 1 × 1 cm graphite electrode is integrated in the center and further additional access points allow fluidic access via insertion of cannulas as well as the insertion of the tailored reference electrode. Three triplicates, each consisting of two microfluidic reactors (anodic and cathodic reactor), fit into a chamber (**c**), which consists of a bottom plate, a frame and a lid. The lid is placed on the anodic compartment so that cultivation can be conducted under anoxic conditions by purging this compartment with gas. In **d** a photograph of a frame can be seen.

In conventional bioelectrochemical systems (BES), it is usually difficult or even impossible to correlate biofilm growth with current density. Multi-parallel analysis of process conditions under laminar flow conditions has also not been possible to date. Therefore, a platform was developed that integrates non-invasive real-time *in situ* 3D imaging based on optical coherence tomography (OCT) with microfluidic flow reactors (Fig. 1). Two reactors were connected in series to form a complete BES. The channel design (S2-3) ensured laminar flow across the electrode. The upstream reactor served as the working electrode and carried a customized Ag/AgCl reference electrode 21 mm downstream of the working electrode. To operate the working electrode as an anode, anoxic conditions were assured in the working electrode chamber by placing it in a sealed polycarbonate enclosure that was continuously purged with 30 mL min^−1^ 80% N_2_/20% CO_2_ gas while the cathode chamber was kept under oxic conditions. The enclosure was designed to hold three specimens, each consisting of an anode and a cathode reactor (S4). Blunt cannulas (B. Braun, Melsungen, Germany), Luer-Lock connectors and silicone tubing (1 mm inner diameter; Carl Roth, Karlsruhe, Germany) were used to connect the corresponding microfluidic electrode reactors to each other and to the media supply. The electrodes were connected to a potentiostat (BioLogic VMP-300, Seyssinet-Pariset, France) via a silver foil (0.1 mm; Chempur, Karlsruhe, Germany). The reactor was characterized by chronoamperometric analyses with an applied potential of 0 V (vs. SHE). To enable (semi-)automated OCT imaging, a gantry robot (DLE-RG-0003, igus® GmbH, Cologne, Germany) was developed that can image up to six polycarbonate housings (Fig. 1a). OCT is a non-invasive imaging technique that produces cross-sectional images of scattering materials such as biofilms. The images could be taken through the polycarbonate lid. Therefore, the OCT probe head was mounted on the gantry robot. This allowed monitoring of biofilm development on all working electrodes with high reproducibility and low operational requirements throughout the experiment. The temperature of the entire workspace was set to 30 °C for the experiments presented in this study.

## 3 Results & discussion

### 3.1 Quantitative analysis of electroactivity of single and mixed-species biofilms of *Shewanella oneidensis* and *Geobacter sulfurreducens*

**Fig. 2:**
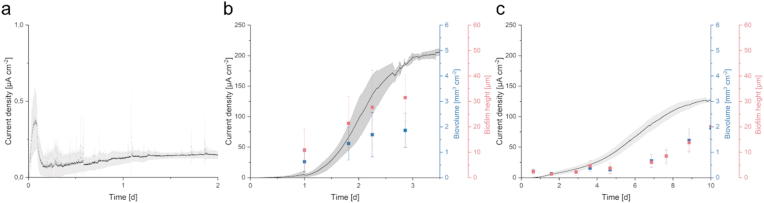
**Comparison of growth in a microfluidic microbial electrolysis cell (MEC) of *S. oneidensis* (a), *G. sulfurreducen*s (b) and a *S. oneidensis*/*G. sulfurreducens* co-culture (c).** The current density was analyzed until it reached a plateau. Biofilm height and biovolume were examined using optical coherence tomography (OCT). Error bars represent the standard deviation from individual replicates (n = 3).

The aim of this study was to analyze how interspecies dependence or even cooperation would affect the development of anodic biofilms. The model community for this endeavor consisted of the two model organisms for electrode interaction *S. oneidensis* and *G. sulfurreducens*. Interdependence can be easily achieved by feeding the community with lactate, which is converted to acetate by *S. oneidensis*. Previous studies have shown that lactate cannot be metabolized by the wild type of *G. sulfurreducens* and that only long-term evolution in the laboratory can lead to an adaptation to lactate as a carbon and electron source [52]. In contrast, acetate is the preferred substrate for *G. sulfurreducens*. In order to observe the difference in biofilm development, individual cultures of the two organisms were first analyzed. During the inoculation of *S. oneidensis*, a peak in current density of approximately 360 ± 170 nA cm^−1^ was observed. Thereafter, the current density dropped sharply and leveled off at 150 ± 20 nA cm^−1^ after 26 h (Fig. 2a). It was not possible to collect biofilm data as the ability of *S. oneidensis* to form anodic biofilms is rather limited [[53], [54], [55]] and the biofilm thickness was in the lowest range of measurement sensitivity of the OCT device (8 × 8 × 5.5 μm). *G. sulfurreducens* was cultivated directly with acetate as a carbon and electron source. Inoculation of *G. sulfurreducens* initially led to a lag phase in current density for 24 h, followed by a logarithmic growth phase leading to a stationary plateau of approximately 200 ± 10 μA cm^−2^ after 72 h (Fig. 2b). It is noteworthy that after 24 h the current density was still comparatively low at 4 ± 3 μA cm^−2^, but the biofilm height and biovolume already reached values of 10.8 ± 8.4 μm and 0.6 ± 0.4 mm^3^ cm^−2^, respectively, which corresponds to about one third of the values after 72 h, when the current density reached its plateau. A linear correlation between biovolume and current density could therefore not be observed over the course of the experiment.

The results regarding current density are in line with previously published literature on both model organisms. *S. oneidensis* is a poor anodic biofilm former, that generally relies heavily on mediated electron transfer (MET) [53,56]. However, in a flow-through system, MET cannot contribute to power generation because shuttle molecules such as riboflavin are constantly washed out. This leaching represents an additional metabolic burden for the organism [57]. *G. sulfurreducens* formed conductive anodic biofilms with a thickness of more than 30 μm after 72 h and produced 1333 times the current density compared to *S. oneidensis*. Furthermore, the *G. sulfurreducens* biofilm required more time to reach a current density plateau, which could be explained by a slower division rate and the development of more complex biofilm structures [33,[58], [59], [60]].

Inoculation of *S. oneidensis* and *G. sulfurreducens* (in a ratio of 10:1) with lactate as the sole carbon and electron source in the medium led to remarkable differences compared to the single culture experiments. The lag phase in the current density of the co-culture was about 24 h and a current density plateau of 130 ± 3 μA cm^−2^ was reached after 9–10 days. Biofilm height and biovolume remained rather low during the first 5 days and increased linearly from day 7 until a biofilm height and volume of 20 μm and 2 mm^3^ cm^−2^, respectively, were reached on day 10. While considerable biofilm growth was observed in *G. sulfurreducens* even before a sharp increase in current density, the co-culture lagged behind the development of current density in terms of biofilm growth.

**Fig. 3:**
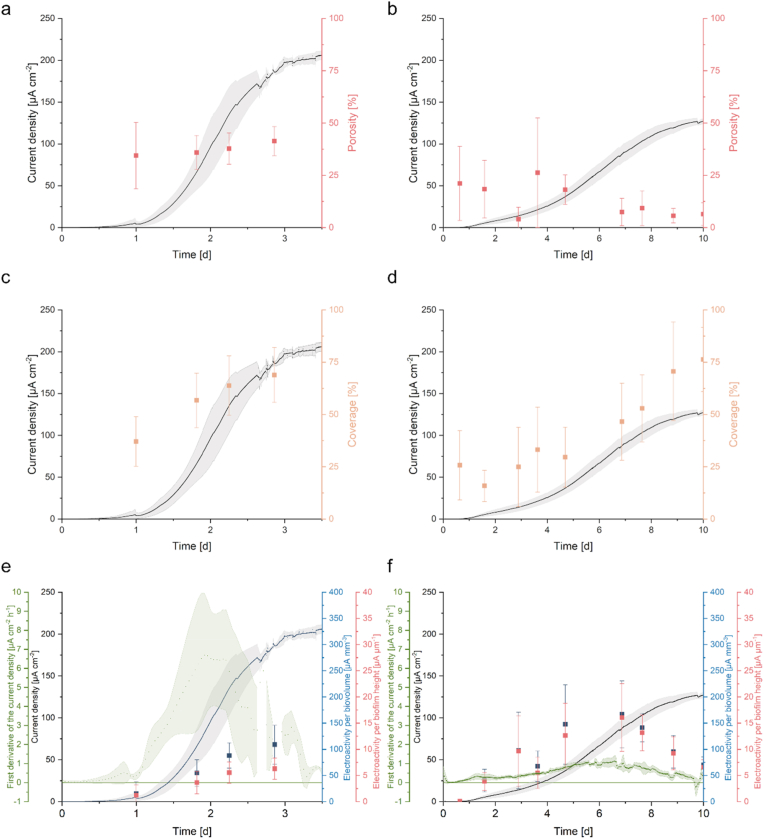
**Biofilm porosity (a-b), coverage (c-d) and the first derivative of current density, electroactivity per biovolume as well as electroactivity per biofilm height per biofilm height (e-f) are plotted over time in comparison to current density.** Error bars represent the standard deviation from individual replicates (n = 3).

Since no biofilm data could be generated for *S. oneidensis*, the behavior of the co-culture can only be compared with *G. sulfurreducens* biofilm data in the following section. Biofilm porosity describes the proportion of voids in the biofilm matrix. The more compact a biofilm is, the fewer voids it has. Fig. 3 a and b show the porosity of the biofilms of *G. sulfurreducens* and the co-culture. While the porosity of the biofilm of *G. sulfurreducens* was about 40 % during the whole experiment, it decreased in the co-culture from about 25 % at the beginning to 8 % at the end of the experiment. In general, the standard deviation becomes smaller with increasing biovolume, as interference signals, which can never be completely avoided in OCT imaging, have less influence.

Surface coverage (Fig. 3c–d) increased with current density, reaching 75 % coverage at maximum current density for both *Geobacter* alone and co-culture. Fig. 3 e-f show not only the current density, but also electroactivity per biovolume (blue) and electroactivity per biofilm height (red), as well as the first derivative of current density (green; indicating the change in current density per unit time). While in *G. sulfurreducens* the first derivative of the current density shows a maximum on day 2 and the electroactivity increases continuously, the activity of the co-culture behaves similarly to the first derivative of the current density and shows a peak value between day 5 and 7. We define the electroactivity as the current generated per biofilm volume or height. The peak of current development in *G. sulfurreducens* on day 2 correlates with the biofilm growth rate, suggesting that current production at this time appears to be primarily dependent on cell number and that other factors limit electroactivity thereafter. This limitation could, for example, be due to the formation of a vertical proton gradient from the bulk phase to the anode surface. The fact that a similar exponential behavior could not be observed in the current development for the co-culture could be due to a limitation by the lactate conversion rate of *S. oneidensis*. This limitation could lead to a more linear growth behavior. It also seems possible that the late biofilm growth in the co-culture, which does not correspond to the slow development of the current density, is due to the growth of the biofilm on or within inactive cell areas that are not subject to direct cell lysis.

It is possible that a higher supply with electron donor and energy source led to more porous biofilms in *G. sulfurreducens* single cultures. Since the biofilm-forming *G. sulfurreducens* cultures in the single culture experiment are supplied with an excess of acetate via the medium, they presumably tend to grow higher and more porous. This enables better substrate availability and lower pH gradients. The biofilms of the *S. oneidensis/G. sulfurreducens* co-culture are only supplied with lactate. This requires the presence of *S. oneidensis* in an active co-culture to establish cross-feeding, where lactate is converted to acetate, which in turn is accessible to *G. sulfurreducens*. In this case, while lactate is abundant in the reactor, acetate is only produced in close proximity to *S. oneidensis*, forcing *G. sulfurreducens* to grow in close proximity to *S. oneidensis*. This behavior is reflected in current production, biofilm height (Fig. 2 b-c), porosity (Fig. 3 a-b) and electroactivity (Fig. 3 e-f), indicating the dependence of *G. sulfurreducens* on *S. oneidensis* for acetate supply and its consequences for biofilm formation and current density production. While *S. oneidensis* could be advantageous for co-cultures in bioelectrochemical batch experiments due to riboflavin production, this factor is minimized by constant dilution with new medium. Therefore, the current production of the co-culture is lower compared to *G. sulfurreducens* alone.

### 3.2 FISH analysis reveals specific species distribution patterns suggesting also interspecies electron transfer

**Fig. 4:**
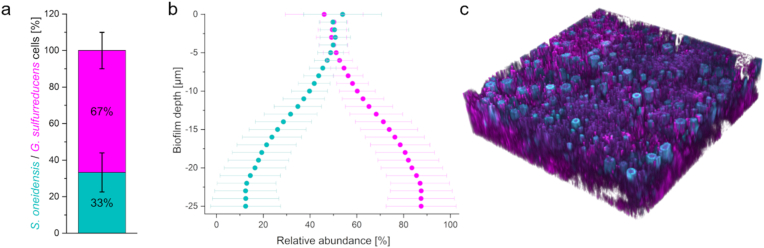
**Spatially resolved fluorescence *in situ* hybridization (FISH) analysis of an *S. oneidensis*/*G. sulfurreducens* biofilm.** In a, the ratio of *S. oneidensis* to *G. sulfurreducens* cells is shown across the entire biofilm. The ratio of the two species plotted against biofilm depth is shown in b. Here, 0 μm is the outer biofilm layer and the further into the biofilm, the more towards the anode (negative numerical values). In c, an exemplary three-dimensional perspective is shown.

To further clarify the spatial distribution and ratio of *S. oneidensis* and *G. sulfurreducens* in the co-culture biofilm, FISH followed by CLSM was performed on a mature biofilm (Fig. 4 a-c). An exemplary 3D image (Fig. 4c) shows that the biofilm consisted of about two-thirds *G. sulfurreducens* cells, which formed a 3D network throughout the biofilm matrix, in which tubular *S. oneidensis* colonies with a diameter of ∼5 μm and a height of ∼10 μm were embedded. These tubes form perpendicular to the anode and are not in direct contact with it (see S5). These data are consistent with the hypothesis above that *G. sulfurreducens* must remain in close proximity to *S. oneidensis* for acetate supply to be as efficient as possible. Each *S. oneidensis* tube is directly surrounded and partially penetrated by *G. sulfurreducens* cells, and we hypothesize that these cells, connected by nanowires, form a conductive network. The tubules are each about 2.5 μm apart (see S5), and indeed the most active *G. sulfurreducens* cells (FISH signal intensity correlates with ribosome abundance) are located directly next to the *S. oneidensis* tubules. The tubules are not in direct contact with the anode surface and mediated electron transfer (MET) is expected to be negligible, as *G. sulfurreducens* is shown to not rely on secreted shuttle molecules for extracellular electron transfer [61,62] and riboflavin secreted by *S. oneidensis* presumably plays only a minor role in flow-through systems and appears mainly as cofactor on the cell surface [63]. Of note, although riboflavin seems to be excreted at least by some *Geobacter* species, it seems to be directly bound to outer membrane cytochromes at least in *G. sulfurreducens* [[64], [65], [66]]. Therefore, electron shuttling was so far not shown to play a role in anode reduction by this organism. Hence, as (I) *G. sulfurreducens* does not release substantial amounts of riboflavin into the medium, as (II) both *G. sulfurreducens and S. oneidensis* bind at least under flow-through conditions riboflavin mostly to outer membrane cytochromes and as (III) the flow through will likely lead to a constant wash out of unbound riboflavin, it seems likely that *S. oneidensis* relies on the conductive network of *G. sulfurreducens* for electron transport. Based on these findings, we hypothesize a direct interspecies electron transfer (DIET) from *S. oneidensis* to *G. sulfurreducens*. To our knowledge, DIET between *Geobacter* and *Shewanella* has not yet been demonstrated, but that conductive pili are not necessary for this has already been shown in *Geobacter* co-cultures [67]. Thus, *G. sulfurreducens* is not only dependent on cross-feeding by *S. oneidensis*, but conversely, *S. oneidensis* seems also dependent on *G. sulfurreducens* for extracellular electron transfer to the anode. Nevertheless, future studies need to provide definitive evidence for the latter, e.g. by performing local cell-cell interaction studies in which only *G. sulfurreducens* cells are attached to a microelectrode and individual *S. oneidensis* cells are brought into contact with these cells.

In addition, the ratio of *S. oneidensis* to G*. sulfurreducens* was determined (Fig. 4a) and resulted in a percentage ranging from 33 ± 10.7 % to 67 ± 10 %. Since these data were obtained by pixel counting within the binarized CLSM images, the actual number of *G. sulfurreducens* cells could be even higher due to their smaller cell size, i.e. fewer pixels per cell.

Looking at the theoretical energy yields of the two organisms, this seems counterintuitive. *S. oneidensis* consumes lactate and produces acetate and carbon dioxide. *Geobacter* completely oxidizes acetate to CO_2_. For the sake of simplicity, it seems appropriate to compare the energy gain for the two organisms with the anaerobic electron acceptor fumarate, which at +33 mV actually has a similar redox potential to the anode used here.

lactate^−^ + 2 fumarate^−^ + H_2_O → acetate^−^ + CO_2_ + 2 succinate^−^ ΔG°’ = -173.11 kJ mol^−1^

acetate^−^ + 4 fumarate^−^ + H^+^ + 2H_2_O → 2 CO_2_ + 4 succinate^−^ ΔG°’ = -232.48 kJ mol^−1^

Assuming a necessary energy input of 80 kJ/mol ATP under reversible cellular conditions, *Geobacter* would gain 2.9 and *Shewanella* 2.2 ATP. Theoretically, both organisms could therefore occur in almost identical quantities. However, *S. oneidensis* is unable to thrive via oxidative phosphorylation using lactate as a carbon source, and the one ATP molecule per lactate consumed, obtained via substrate-level phosphorylation from the conversion of acetyl-CoA to acetate, appears to be most important. In other words, the energy fraction that could be obtained via oxidative phosphorylation is not efficiently utilized for ATP production for some metabolic reason. In this case, the 1:3 ratio observed in fluorescence microscopy would indeed be reflected by the ATP gain of the two organisms.

If the ratio of *S. oneidensis* to *G. sulfurreducens* is plotted against biofilm depth (Fig. 4b), it can be seen that in the upper biofilm layers (0 to −5 μm) the abundance of both organisms is equally distributed. With increasing proximity to the anode, this balance shifts in favor of *G. sulfurreducens*, until at a depth of −25 μm about 87.5 ± 15% of the organisms present are *G. sulfurreducens*. Hence, final stages of electron transfer seem to be almost completely conducted using the *Geobacter* machinery.

### 3.3 Cell viability is highest in the biofilm center

**Fig. 5:**
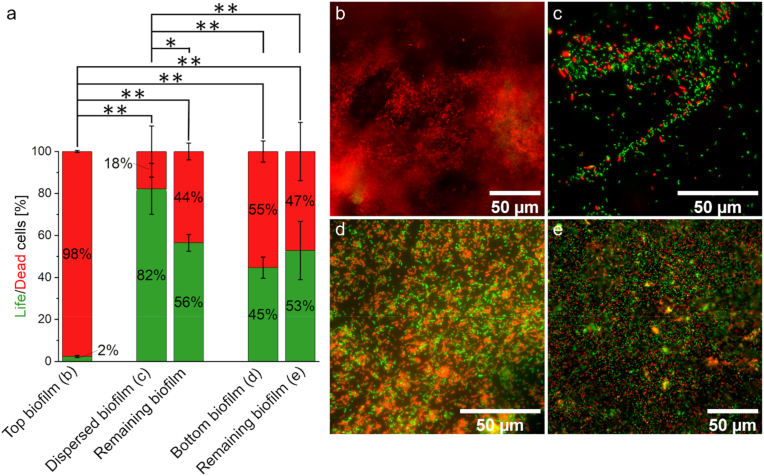
**Life/Dead staining of *S. oneidensis*/*G. sulfurreducens* co-coltures on anodes from microfluidic microbial electrolysis cells (MECs).** In **a** the percentage distribution of living and dead cells is shown, and in **b-e** representative microscope images of dead (red) and living (green) cells in an overlay image can be seen. Two anodes were examined. For both anodes, the biofilm was first examined from above (‘top biofilm’; **b**). The biofilm was then rinsed off with saline solution and a pipette from one anode and the dispersed biofilm was examined (‘dispersed biofilm’; **c**). From the other anode, the biofilm was removed with tweezers and the biofilm was examined from below (‘bottom biofilm’; **d**) and the remaining biofilm on the anode (‘remaining biofilm’; **e**). Error bars of the first column (top biofilm) in **a** represent the standard deviation from individual replicates (n = 2). The error bars of all other columns (upper biofilm) represent the standard deviation of technical replicates (n_dispersed biofilm_ = 7 and n_remaining biofilm_ = 2; n_bottom biofilm_ = 9 and n_remaining biofilm_ = 7). Asterisks represent significant differences (unpaired *t*-test: * = p < 0.05; ** = p < 0.01). (For interpretation of the references to colour in this figure legend, the reader is referred to the Web version of this article.)

As mentioned above, several studies have attempted to define active zones within anode biofilms and have often come to different conclusions. Under the strictly controlled conditions prevailing here, the results of fluorescence microscopy indicate that most activity is found in the center of the biofilm. Fig. 5a shows the ratio of living to dead cells at different locations of the biofilm and b - e show exemplary images at the corresponding locations. While the outermost layer of the biofilm (b) consisted almost exclusively of dead cells, the biofilm layers near the anode surface showed a viability of about 50% (d-e). The biofilm dispersion (c), which represents a cross-section of the entire biofilm from the top layer to the anode surface, had a viability of 82 ± 12 %. Considering that the outer and innermost layers have a viability of 2 ± 0.5 % and 50 %, respectively, this means that the viability of the cells in the center of the biofilm must have been even higher than 82 ± 12 %. Since there are (at least) two opposing gradients within the biofilm (the lactate concentration decreases towards the anode, while the electrical biofilm resistance increases with increasing distance from the anode and thus hinders electron transport [68]) it can be assumed that the most viable layers are located somewhere inside the central biofilm.

## 4 Conclusion

In this study, a novel automated platform for microfluidic (electroactive) biofilm cultivation and analysis was used to study anode biofilms of *S. oneidensis* and *G. sulfurreducens*. In co-culture, *G. sulfurreducens* is dependent on cross-feeding by the lactate-metabolizing *S. oneidensis*, while in turn *S. oneidensis* might utilize the conductive biofilm matrix developed by *G. sulfurreducens*, facilitating DIET to the anode. *S. oneidensis* forms tubular structures within the *G. sulfurreducens* matrix, without direct contact with the anode surface, and the co-culture forms dense biofilms, possibly due to codependency. These results indicate that the architecture of the exoelectrogenic biofilm dynamically adapts to the existing environment. They also provide evidence that DIET can be easily achieved in biofilm systems, as access to the nanowire-based electron transfer network seems to be possible.

Furthermore, the developed platform can be used to study almost any combination of materials and organisms for their time-resolved effect on biofilm formation with high throughput, versatility and reproducibility.

## CRediT authorship contribution statement

**Edina Klein:** Writing – original draft, Visualization, Validation, Software, Methodology, Investigation, Formal analysis, Data curation, Conceptualization. **René Wurst:** Writing – original draft, Visualization, Validation, Methodology, Investigation, Formal analysis, Conceptualization. **David Rehnlund:** Conceptualization. **Johannes Gescher:** Writing – review & editing, Validation, Supervision, Resources, Project administration, Funding acquisition, Conceptualization.

## Declaration of competing interest

The authors declare that they have no known competing financial interests or personal relationships that could have appeared to influence the work reported in this paper.

## Data Availability

Data will be made available on request.
