# Supplementary material for: Elucidating the development of cooperative anode-biofilm-structures
Source: Biofilm. 2024 Mar 25;7:100193. doi: 10.1016/j.bioflm.2024.100193 (PMC11004076; doi:10.1016/j.bioflm.2024.100193)
Supplement: Multimedia component 1 [file mmc1.docx]

**SUPPLEMENTARY INFORMATION**

Supporting and additional information of our findings. This file contains images and data which are meant to give a better idea of our experiment and findings as well as results of side and evaluating experiments.

**Journal name:** Biofilm

**Article title:** Elucidating the development of cooperative anode-biofilm-structures

**S1 The necessary steps to perform the automated fluorescence *in situ* hybridization (FISH) protocol are listed in this table**. RT = room temperature.

| **Work step** | **Solution** | **Flow rate**  *[µL min^‑1^]* | **Pumping time**  *[min]* | **Incubation time**  *[min]* | **Temperature**  *[°C]* |
| --- | --- | --- | --- | --- | --- |
| Fixation | 4 % formaldehyde in PBS (137 mM NaCl, 2.7 mM KCl, 10.  mM Na_2_HPO_4_, 1.8 mM KH_2_PO_4_; v/v) | 67 | 35 | - | RT^a^ |
| Permeabilization | 0,1 M HCl | 67 | 29 | - | RT^a^ |
|  |  | - | - | 10 | RT^a^ |
| Wash | ddH_2_O | 67 | 29 | - | RT^a^ |
| Hybridization I | 13.5 µL mL^‑1^ 50 ng µL^‑1^ SHEW227 (Huggett et al., 2008) probe in 40 % hybridization buffer (0,9 M NaCl, 20 µL mL^‑1^ 1 M TrisHCl pH 8 (v/v), 0.01 % SDS (w/v), 40 % formamide (v/v)) | 67 | 29 | - | 48 |
|  |  | - | - | 90 | 48 |
| Wash | Washing buffer 40% (46 mM NaCl, 20 µL mL^‑1^ 1 M TrisHCl pH 8 (v/v), 0.01 % SDS (w/v)) | 67 | 29 | - | 48 |
| Hybridization II | 13.5 µL mL^‑1^ 50 ng µL^‑1^ SHEW227 (Richter et al., 2007) probe in 40 % hybridization buffer (0,9 M NaCl, 20 µL mL^‑1^ 1 M TrisHCl pH 8 (v/v), 0.01 % SDS (w/v), 40 % formamide (v/v)) | 67 | 29 | - | 48 |
|  |  | - | - | 90 | 48 |
| Wash | Washing buffer 20% (43 mM NaCl, 20 µL mL^‑1^ 1 M TrisHCl pH 8 (v/v), 0.01 % SDS (w/v)) | 67 | 29 | - | 48 |
| Wash | SSC (3 M NaCl, 0,3 M trisodium citrate dihydrate, pH adjusted to 6 with 7 M HCl) | 67 | 29 | - | RT^a^ |
| Wash | PBS | 67 | 29 | - | RT^a^ |
| Counterstaining | 1 µg mL^-1^ DAPI | 67 | 29 | - | 48 |
|  |  | - | - | 5 | 48 |
| Embedding | Embedding buffer (785 µL mL^‑1^ CitiFluor^TM^ AF3 (Science Services GmbH, München, Germany), 143 µL mL^‑1^ VECTASHIELD^®^ Antifade Mounting Medium (vector Laboratories, Newark, California, United States), 71 µL mL^‑1^ PBS) | 67 | 29 | - | RT^a^ |
| Storage | - | - | - | ∞ | 4 |

Fluorescence *in* *situ* hybridization (FISH) was performed as an endpoint analysis after 10 days of cultivation. An automated FISH procedure was applied and the necessary steps are listed in S1. The entire FISH procedure was conducted within the microfluidic reactor by pumping the individual solutions through the biofilm directly after the end of the experiment. To this end, all solutions were connected to active 2/2 normally closed valves (SMV-2R-BN1F; Takasago Kōryō Kōgyō, Tokyo, Japan). The valves were steered using a custom-made control unit. Via Y-branches, all solutions were joined in a single outlet tube, which was attached to an REGLO ICC peristaltic pump (ISMATEC Industry Solutions GmbH, Grevenbroich, Germany). The solutions were supplied to the microfluidic chip at 4 ml h^‑1^.

To further clarify the spatial distribution and ratio of *S. oneidensis* and *G. sulfurreducens* in the mature coculture biofilm, FISH images were taken by means of CLSM. An exemplary 3D image is shown in Fig. 4c and the individual slices can be seen in S5 (*S. oneidensis* in cyan and *G. sulfurreducens* in magenta) and S6 (DNA stained with DAPI).


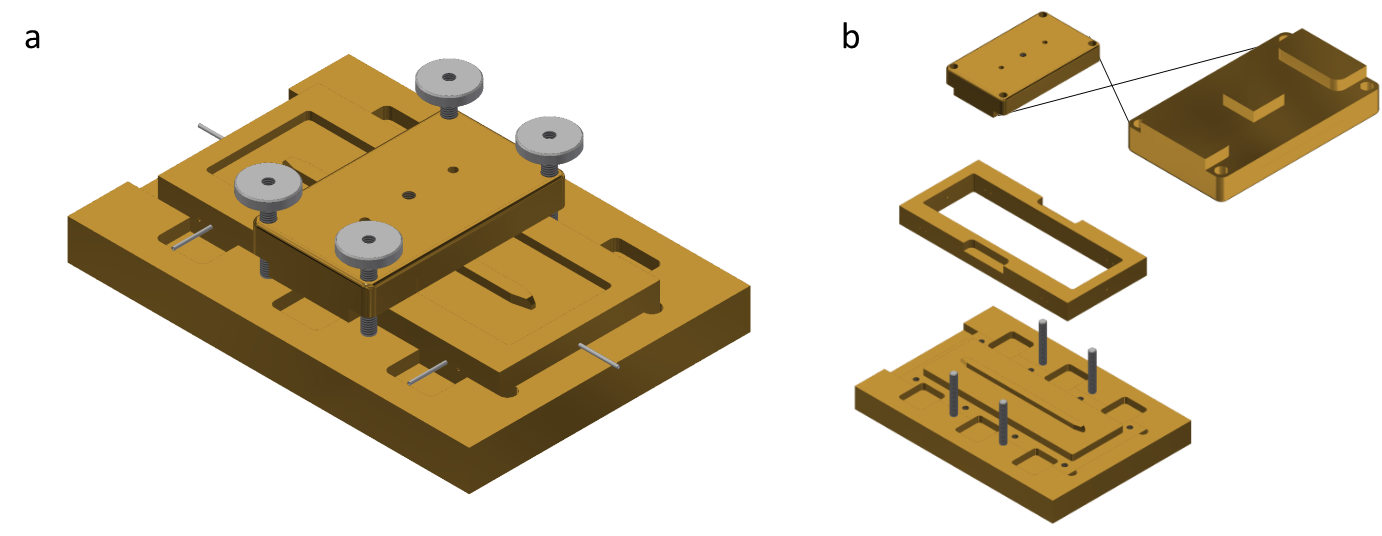


**S2** **Casting mold for the fabrication of tailor-made microfluidic bioelectrochemical reactors.** A fully assembled mould is shown in **a**. By inserting cannulas, four in this figure, accesses can be obtained. The mould consists of a base plate with the respective channel geometry, a frame with holes for inserting cannulas, and a structure for recessing a 1 cm^2^ section for later insertion of the electrode **(b)**.

The polydimethylsiloxane (PDMS) microfluidic reactors with a straight channel design were produced as recently described (Hansen et al., 2019). However, the casting chamber was extended so that a 1 x 1 cm piece can be inserted (S1). The channel design (S2-3) ensured laminar flow over the electrode. Two reactors were connected in series to form a complete BES (S4). The upstream reactor served as working electrode and carried a tailor-made Ag/AgCl reference electrode 21 mm downstream of the working electrode. For operating the working electrode as anode, anoxic conditions were established in the working electrode compartment, by placing it in a sealed polycarbonate housing that was continuously purged with 30 mL min-^‑1^ 80% N_2_/ 20% CO_2_ gas, while the cathode compartment was kept under oxic conditions. The housing was designed to hold a triplicate, each consisting of an anode and cathode reactor.


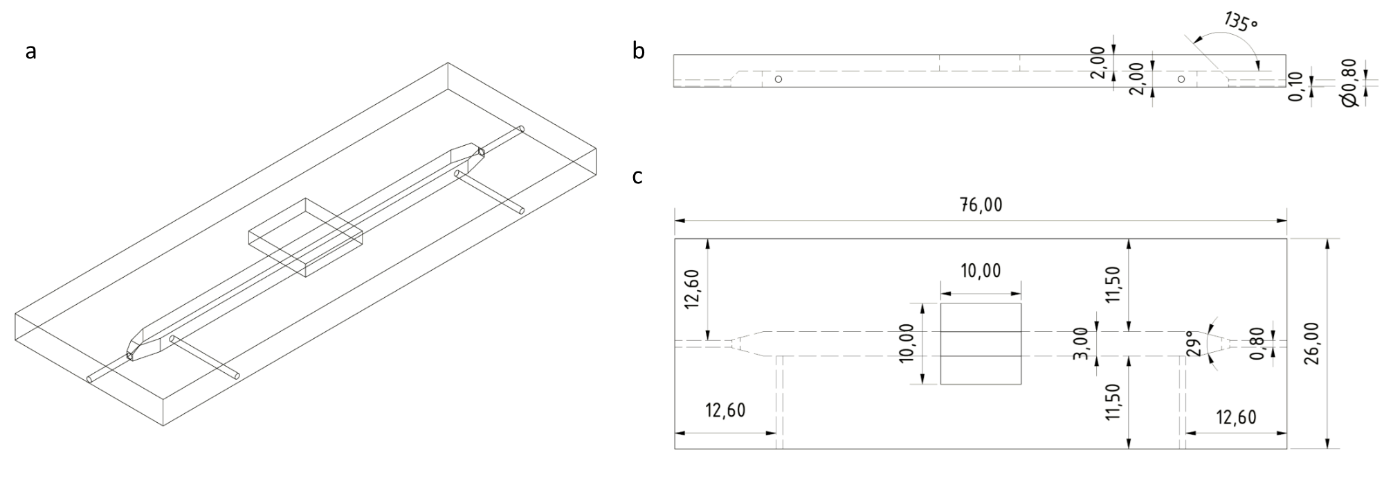


**S3** **Technical drawings of a microfluidic bioelectrochemical reactor.** A three-dimensional illustration of an exemplary microfluidic reactor is shown **(a)**, as well as the dimensions of the reactor from the side **(b)** and from the top **(c)**.


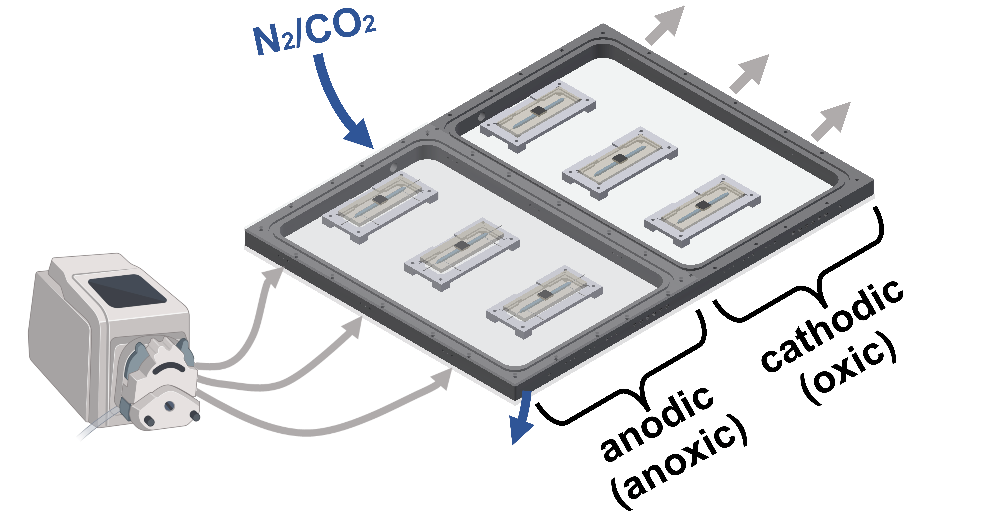


**S4** **Schematic illustration of a frame that provides space for a triplicate of bioelectrochemical microfluidic experiments.** One frame consists of a bottom plate, a frame and a lid. The lid is placed on the anodic compartment so that the cultivation can be conducted under anoxic conditions by purging this compartment with gas. Cannulas inserted into the frame provide the interface for the supply of medium as well as for the outlet.


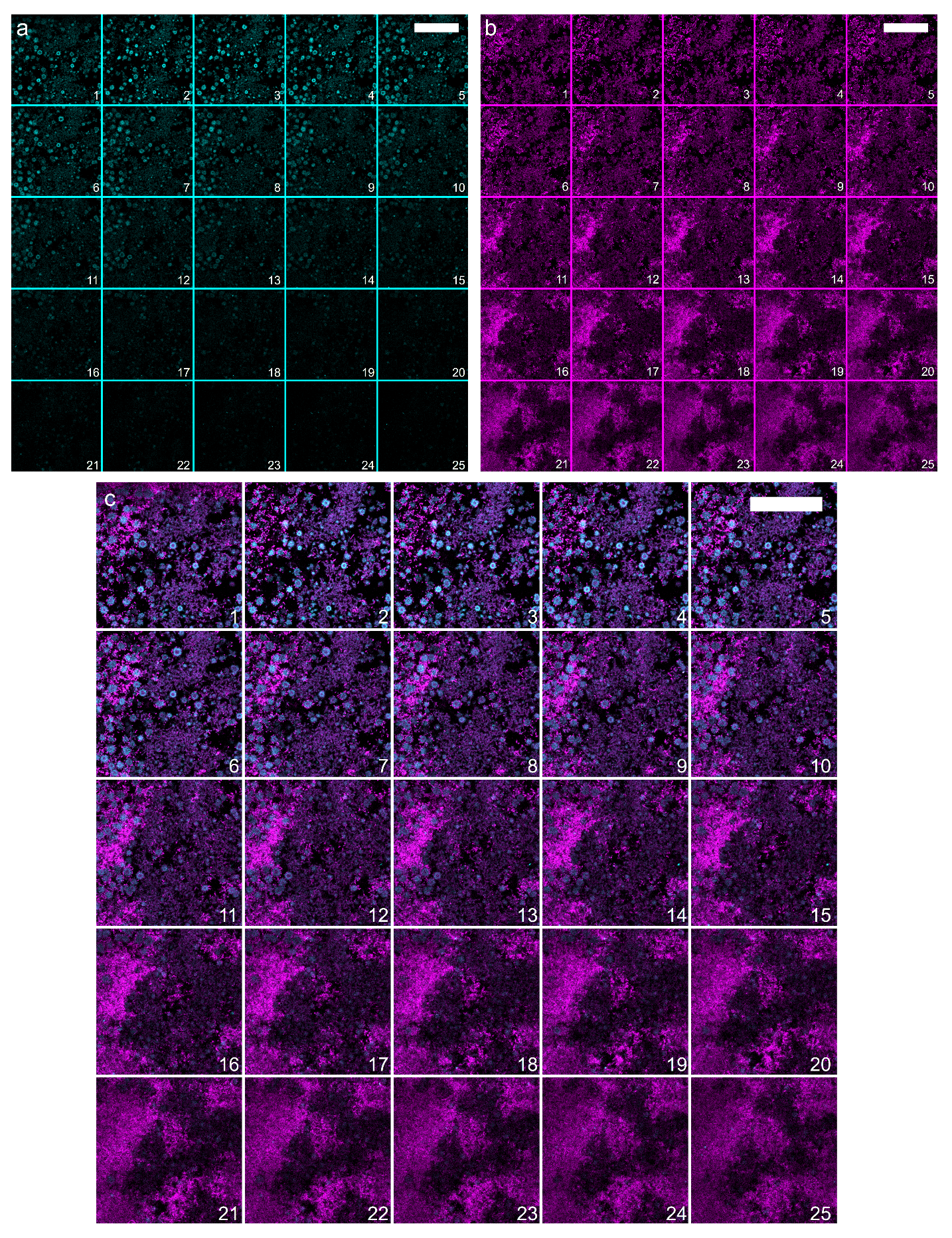


**S5** **Spatially resolved fluorescence *in situ* hybridization (FISH) analysis of a *S. oneidensis* / *G. sulfurreducens* biofilm.** The individual slices of the three-dimensional view from Figure 5c are shown. *S. oneidensis* is shown in **a**, *G. sulfurreducens* in **b** and the overlay in **c**. The white bar represents 50 µm and images were taken at 1 µm intervals.


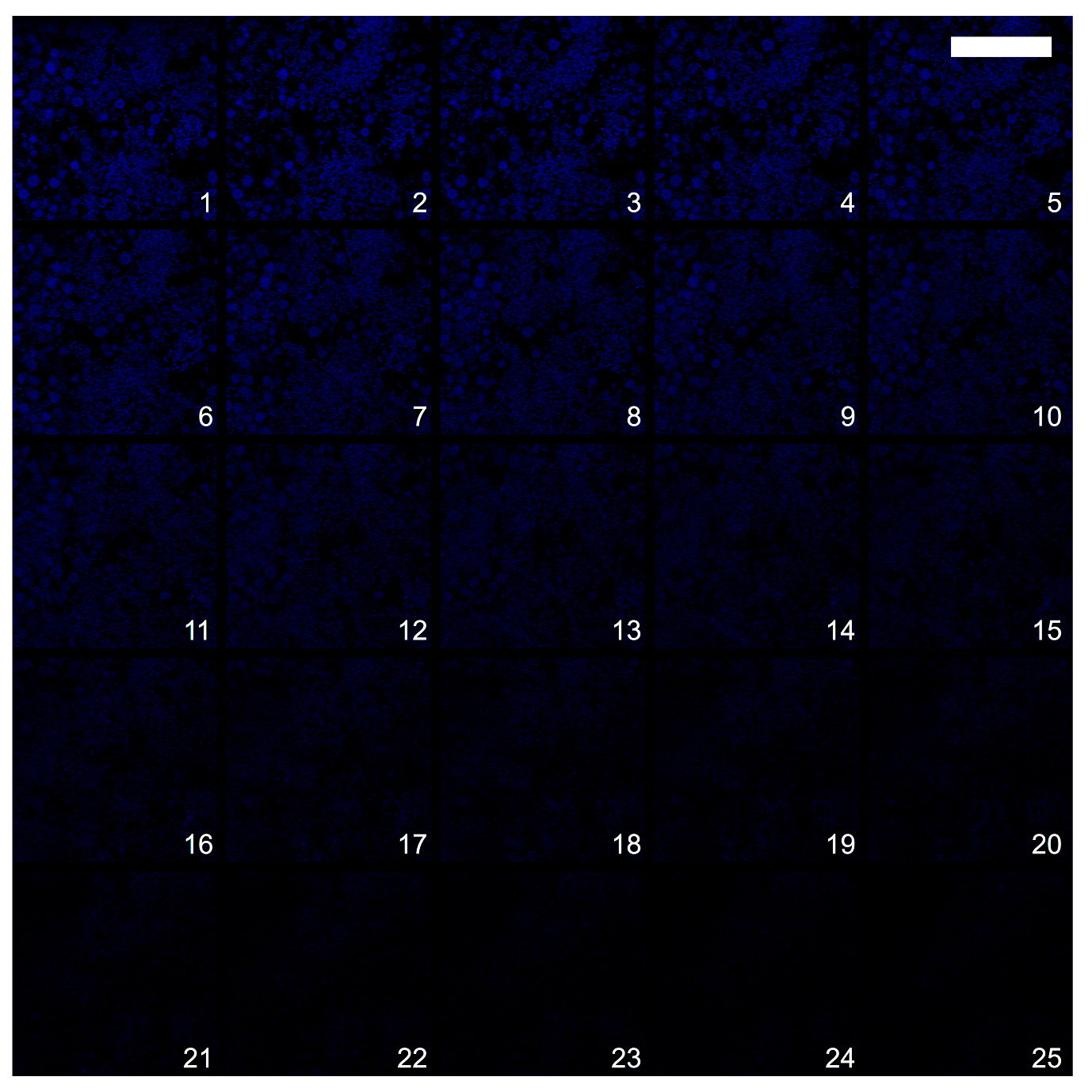


**S6 Spatially resolved DAPI staining of a *S. oneidensis / G. sulfurreducens* biofilm from a fluorescence *in* *situ* hybridization (FISH) analysis.** The individual slices of the 3D view from figure 5c are shown. The white bar represents 50 µm and the images were taken at 1 µm intervals.

**References**

Hansen SH, Kabbeck T, Radtke CP, Krause S, Krolitzki E, Peschke T, Gasmi J, Rabe KS, Wagner M, Horn H, Hubbuch J, Gescher J, Niemeyer C. Machine-assisted cultivation and analysis of biofilms. Sci Rep 2019;9:8933. https://doi.org/10.1038/s41598-019-45414-6.

Huggett MJ, Crocetti GR, Kjelleberg S, Steinberg PD. Recruitment of the sea urchin *Heliocidaris* *erythrogramma* and the distribution and abundance of inducing bacteria in the field. Aquatic Microbial Ecology 2008;53:161–71. https://doi.org/10.3354/AME01239.

Richter H, Lanthier M, Nevin KP, Lovley DR. Lack of electricity production by *Pelobacter* *carbinolicus* indicates that the capacity for Fe(III) oxide reduction does not necessarily confer electron transfer ability to fuel cell anodes. Appl Environ Microbiol 2007;73:5347–53. https://doi.org/10.1128/AEM.00804-07.
